# Genomics and transcriptomics of the Chinese mitten crabs (*Eriocheir sinensis*)

**DOI:** 10.1038/s41597-023-02761-4

**Published:** 2023-11-30

**Authors:** Nan Yang, Wenjing Li, Wenrong Feng, Meiyao Wang, Aimin Liu, Yongkai Tang, Shengyan Su

**Affiliations:** 1 Wuxi Fisheries College, Nanjing Agricultural University, Wuxi, 214081 PR China; 2 Jiangsu Haorun Biological Industry Group Co., Ltd, Taizhou, 225309 China; 3 Key Laboratory of Integrated Rice-Fish Farming Ecology, Ministry of Agriculture and Rural Affairs, Freshwater Fisheries Research Center, Chinese Academy of Fishery Sciences, Wuxi, 214081 China

**Keywords:** Ichthyology, Gene expression

## Abstract

To gain a deeper understanding of the genetic factors influencing the growth and development of Eriocheir sinensis, a well-known species of hairy crab found in Yangcheng Lake, this study focused on the de novo genome and full-length transcriptome information of the selected subjects. Specifically, Yangcheng Lake hairy crabs were chosen as the experimental samples. Initially, a genome analysis was performed, resulting in the identification of gene fragments with a combined length of 1266,092,319 bp. Subsequently, a transcriptome analysis was conducted on a mixture of tissues from four different sites, namely muscle, brain, eye, and heart, to further investigate the genetic characteristics at the transcriptome level. The Pacific Biosciences (Pacio) single-molecule real-time sequencing system generated a total of 36.93 G sub-fragments and 175,90041 effective inserts. This research contributes to the indirect comprehension of genetic variations underlying individual traits. Furthermore, a comparison of the obtained data with relevant literature emphasizes the advantages of this study and establishes a basis for further investigations on the Chinese mitten crab.

## Background & Summary

Chinese eriocheir crabs, scientifically classified as members of the genus eriocheir within the Crustacean Decapoda family, possess a diverse array of mineral elements, fatty acids, amino acids, and other essential nutrients^1^. These crabs are extensively consumed in eastern China and enjoy widespread popularity throughout the nation, leading to the establishment of processing techniques centered around the utilization of hairy crabs as the primary product. Consequently, in recent years, hairy crabs have emerged as a freshwater species of considerable market economic worth.The presence of diverse types of hairy crabs, including Yangcheng Lake hairy crabs and Taihu hairy crabs, has led to the establishment of distinct and comprehensive business models. Nevertheless, the inadequate protection of the Chinese eriocheir crab seed industry, along with the disorderly introduction and indiscriminate release of seedlings, has resulted in the mixing of germplasm resources and significant variations in individual growth and development. Consequently, a severe degradation has transpired, impeding the advancement of high-quality Chinese eriocheir crab products^2^. In recent years, molecular biology techniques have been employed to selectively breed alleles or genotypes possessing advantageous traits in the Chinese mitten crab. For instance, Xiong Liangwei^3^ utilized 60 microsatellite markers to identify polymorphisms in the parents and offspring of the F1 population of Chinese mitten crab. This analysis encompassed the determination of parent-offspring genetic segregation patterns and linkage relationships, ultimately leading to the successful construction of a genetic linkage map specific to the Chinese mitten crab. The construction of a genetic linkage map is a crucial stage in the advancement of molecular markers for facilitating marker assisted breeding of Eriocheir sinensis.

While there exists a substantial body of research on the Chinese eriocheir crab, there is a dearth of studies focusing on genome-wide selective breeding. It is widely recognized that the impact of genes on offspring cannot be disregarded. The proper expression of the dominant genome stands as a crucial prerequisite for attaining superior product quality. To enhance the market-driven impact of hairy crabs and facilitate the advancement of the national economy, a comprehensive comprehension of their life cycle and dietary preferences, along with the implementation of scientific breeding practices, balanced nutrition, and a thorough transcriptome analysis, are imperative. The documentation of the hairy crab genome is of utmost importance, as it serves as a fundamental resource for comprehending biological mechanisms. Transcriptome analysis serves as a valuable tool for enhancing comprehension of the physiological and biochemical attributes of animals, consequently enhancing the overall quality of human existence. By providing insights into cell response, gene function, and evolution, transcript data offers a direct means of comprehending diverse biological processes at the molecular level^4^. Consequently, transcriptome analysis aids in the comprehensive understanding of genetic mechanisms governing internal cell growth, development, and immune regulation^5^ in animals, thereby facilitating a deeper comprehension of the intricate nature of genes. The advent of RNA-seq technology has significantly advanced the utilization of fish transcriptome research^6,7^. Short-read transcriptome sequencing has become a prevalent approach for characterizing gene expression levels, enabling the acquisition of transcripts from both model and non-model organisms using second-generation sequencing platforms^8^. In the case of hairy crabs, a freshwater species of economic importance, the application of RNA-seq technology can offer valuable assistance and guidance for crab analysis and research. The groundbreaking advancements in the domain of ichthyology have facilitated our ability to undertake investigations on crustaceans. Earlier investigations have revealed that the second-generation sequencing technology falls short in acquiring precise and comprehensive transcript data, with the read fragment length exceeding that of eukaryotic mRNA. Conversely, the third-generation sequencing technology, commonly referred to as SMRT^9^, effectively addresses this issue by accommodating shorter fragment lengths. Furthermore, the utilization of SMRT sequencing technology enables the direct acquisition of unspliced complete transcripts, thereby facilitating practical applications. Moreover, an essential advantage of SMRT lies in its ability to complement gene annotation^10^. Currently, single-molecule real-time long-read sequencing (SMRT) stands as a highly dependable approach for comprehensive cDNA molecular sequencing. Its successful implementation in the analysis of full-length transcriptomes across various organisms, including humans, animals, plants, and insects, has yielded a more accurate representation of the transcriptome information encompassing the entire species sequence^11–15^.

In this study, we initially conducted genome sequencing followed by comprehensive transcriptome analysis of Yangcheng Lake Crab across four distinct tissues utilizing PacBioSMRT sequencing technology. This investigation serves as a fundamental basis for future research endeavors. Additionally, we performed a comparative analysis between the findings of this study and previously published literature. Among them, the functional annotation of transcripts from crabs and the analysis of simple repeat sequences (SSRs) may offer valuable insights for future investigations in other species. The comprehensive understanding of the data in this study can be achieved through comparative analysis, thereby establishing a solid theoretical foundation for the future research and development of the Chinese mitten crab.

## Methods

### Selection and preparation of samples

The crab specimens utilized for RNA extraction and sequencing investigations were maintained under optimal growth conditions. To facilitate the sequencing research, four male crabs were selectively captured from Yangcheng Lake in Suzhou City. Subsequently, each crab was transported to the laboratory and housed within a spacious aquarium. The aquarium’s temperature was consistently maintained at approximately 25 degrees Celsius, while the pH levels were regulated within the range of 6.5 to 7.5. Additionally, a low-intensity light source was continuously provided during the natural light cycle to facilitate the crabs’ acclimation to their new environment.

The crab samples that demonstrated viability after a period of two to three days of acclimation were subjected to aseptic dissection. Prior to dissection under sterile conditions, tricaine mesylate (MS-222) was administered to anesthetize four crab samples for the purpose of facilitating sampling. Subsequently, sterile forceps and scissors were employed to separate the muscle, brain, eyestalk, and heart from the body cavity^16^. These obtained samples were then promptly frozen in liquid nitrogen and stored in a refrigerator set at −80 °C to enable the isolation of total RNA. High quality RNA is the basis for the success of the whole project. In order to ensure the accuracy of the sequencing data, we use advanced equipment of molecular biology. The purity, concentration, and integrity of RNA samples are measured to ensure the use of qualified samples for transcriptome sequencing. After the sample test is qualified, the library is constructed. Subsequently, state-of-the-art molecular biology equipment is employed to evaluate the library’s quality, and the test results must meet the specified criteria before advancing to machine sequencing. Following successful library validation, full-length transcriptome sequencing is conducted using the PacBio instrument, in accordance with the desired data volume.

### RNA Library construction

The tissue samples were extracted from the refrigerator, and the ribonucleic acid was isolated from each tissue using TRIzol reagent as per the manufacturer’s guidelines. The sample concentration was determined using a spectrophotometer and Agilent biometer, while the RNA integrity was calculated using the Agilent 2100 bioanalyzer (Agilent Technologies, Santa Clara, USA). The RNA derived from the identical tissue of the acquired sample was combined to generate an iso-seq library, and the initial strand cDNA was synthesized utilizing the SMARTer, PCR, and cDNA synthesis kit. Following the initial round of cDNA amplification, the BluePippon selection system was employed to segregate the cDNA into distinct size fragments to avoid sequencing. Subsequently, after the library was constructed and subjected to quality control measures, the complete transcriptome sequence comprising three full-length transcripts was acquired through PacBio sequencing.

### Genome assembly

**Fig. 1:**
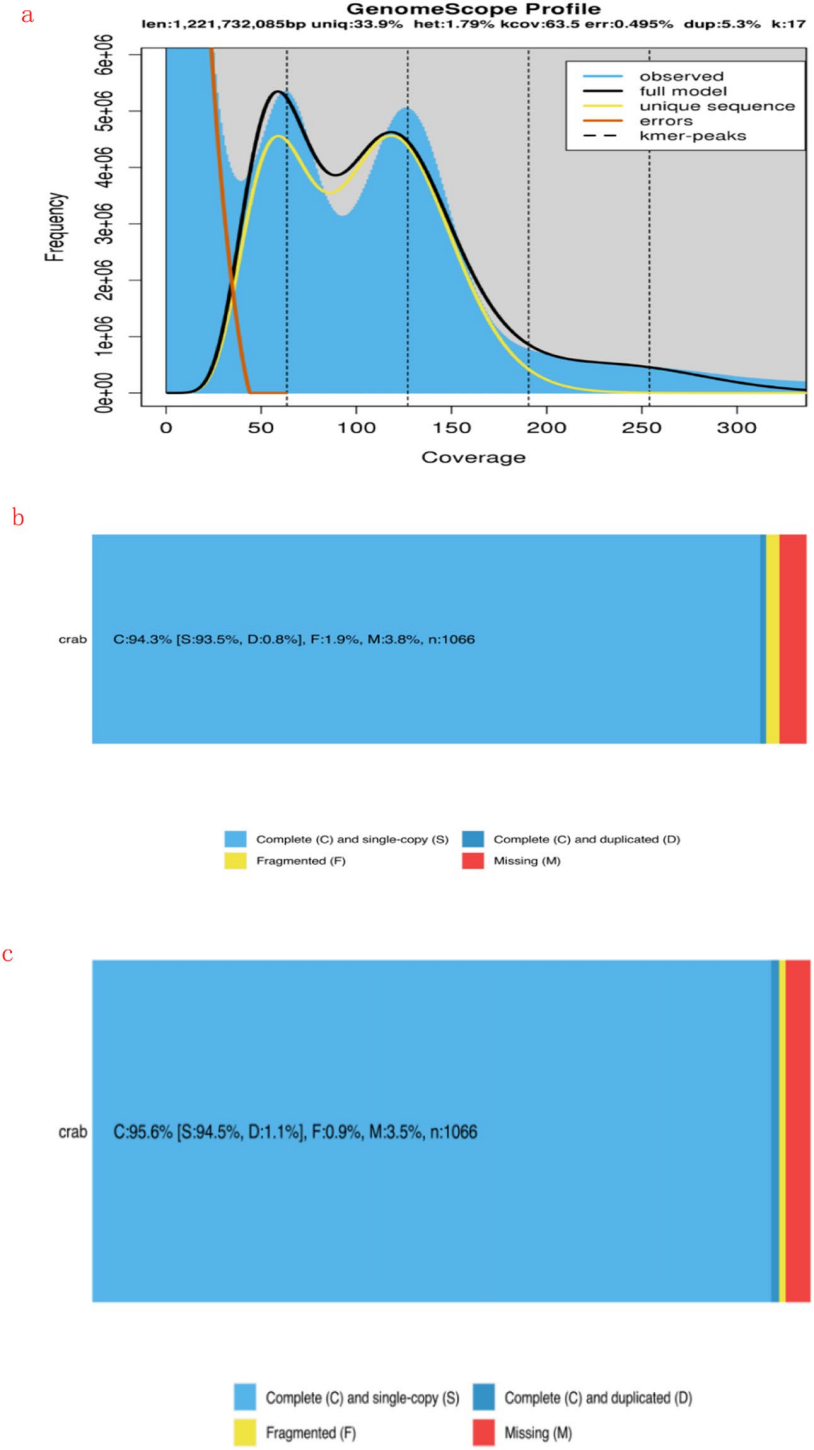
(**a**) K-mer coverage depth; (**b**) Genome BUSCO map of crab after purge; (**c**) Genome BUSCO map of crab after purge; (**d**) BUSCO evaluation diagram of crab final assembly results.

17-mer statistics are performed on the sequenced fastq data by using jellyfish^17^ and then GenomeScope^18^ is used to estimate genome heterozygosity and duplication. We chose a k-mer depth threshold of 100000. We estimated the crab genome size to be 1,221,732,085 bp, heterozygosity to be 1.79%, and duplication to be 66.1% (Fig. 1a). Sequenced by the PacBio SequelII platform, 113,241,560,168 bp of raw data was obtained, approximately (113 G). The statistics of raw data are shown in the table below. Using wtdbg2 software to assemble the PacBio data, the genome size was 1,486,013,762 bp, or about 1.4 G. The large assembly result may be due to the high heterozygosity of the species. The contigN50 was 123,603 bp, and there were 29,073 contig in total. The longest contig was 3,041,852 bp, and the average length of contig was 51,113 bp (Table 1). The original sequencing data of PacBio was compared with the above assembly results to remove redundant parts of the genome. The resulting genome size was 1,263,668,219 bp, or about 1.26 G. contigN50 is 159,691 bp, with a total of 18,479 contig, of which the longest contig is 3,071,904 bp, and the average length of contig is 68,384 bp. < br / > BUSCO^19^ (Benchmarking Universal Single-Copy Orthologs: http://busco.ezlab.org/) Evaluation is to evaluate the integrity of the assembled genome by using a single copy orthologous gene library and combining software such as tblastn and augustus^19^ to evaluate the assembled genome. We evaluated the completeness of 1066 single-copy genes in arthropoda after purge, and obtained that the completeness of crab genome was 94.3% respectively (Fig. 1b). We will use Arrow to conduct a round of self-comparison and error correction of the third-generation data, and then use the second-generation data in the Survey to conduct two rounds of Pilon error correction to obtain the final assembly version, and the final assembly will obtain 6908 scaffscaffold, 1,266,092,319 bp. Contains 18472 contig. The scaffold N50 was 17,444,176 bp and the contig N50 was 159,228 bp. < br / > BUSCO assessed the integrity of the genome at 95.6% (Fig. 1c).

**Table 1:** PacBio data statistics.

Raw data statistics	Species	Total(bp)		MaxLen(bp)	mean length(bp)		subreads mean N50	
	Crabs	113,241,560, 168		2,07,711	13,508		20,314	
**Raw data statistics**	**Species**	**ScaNum**	**CtgNum**	**BaseNu m(bp)**	**MaxCtgLen(bp)**	**MeanCtg Len(bp)**	**ScaN 50**	**Ctg N50**
	crab	6908	18472	1,266,0 92,319	41,692,7 56	183,279	17,44 4, 176	159, 228

### HiC raw data and quality control

The sample cells are fixed with formaldehyde to cross-link DNA to proteins and proteins to proteins. After the cross-linking of the samples was completed, cell lysis was performed, and sample extraction was performed to detect the quality of the samples. After the test is qualified, the Hi-C library preparation process is entered. Chromatin digestion was performed with restriction endonuclides, and the effect of enzyme digestion was measured with samples. After biotin labeling, flat end ligation and DNA purification, Hi-C samples^20^ were prepared, and DNA quality was detected by sampling. After passing the test, enter the standard library construction process. The Hi-C fragment was debiotin, interrupted by ultrasound, repaired by adding base A and sequencing splice to form splice product. The product of the library was then screened and amplified by PCR. The product of library amplification was sampled for “Hi-C fragment junction point quality control test”, and the whole library was prepared after passing the test. After qualified quality control, the constructed library was sequenced by Illumina HiSeq with sequencing strategy PE150. The data obtained by sequencing is the original disembarkation sequence, which will contain sequencing connector sequences and low-quality sequences. In order to ensure the quality of information analysis data, fastp^21^ (software version: 0.20.0; The original sequences were filtered using default parameters), and high-quality Clean Reads were obtained, duplicate Reads were removed, and follow-up analysis was conducted, all of which were based on Clean Reads (Table 2).

**Table 2:** Sequencing data statistics.

Restrction enzyme	Restriction Enzyme cutting site	Total Yield (bp)	Q20%	Q30%	GC%	Total reads
MboI	GATC	288,759,220,191	96.26%	90.85%	43.47%	1,929,604,516

### HiC assisted genome assembly

**Fig. 2:**
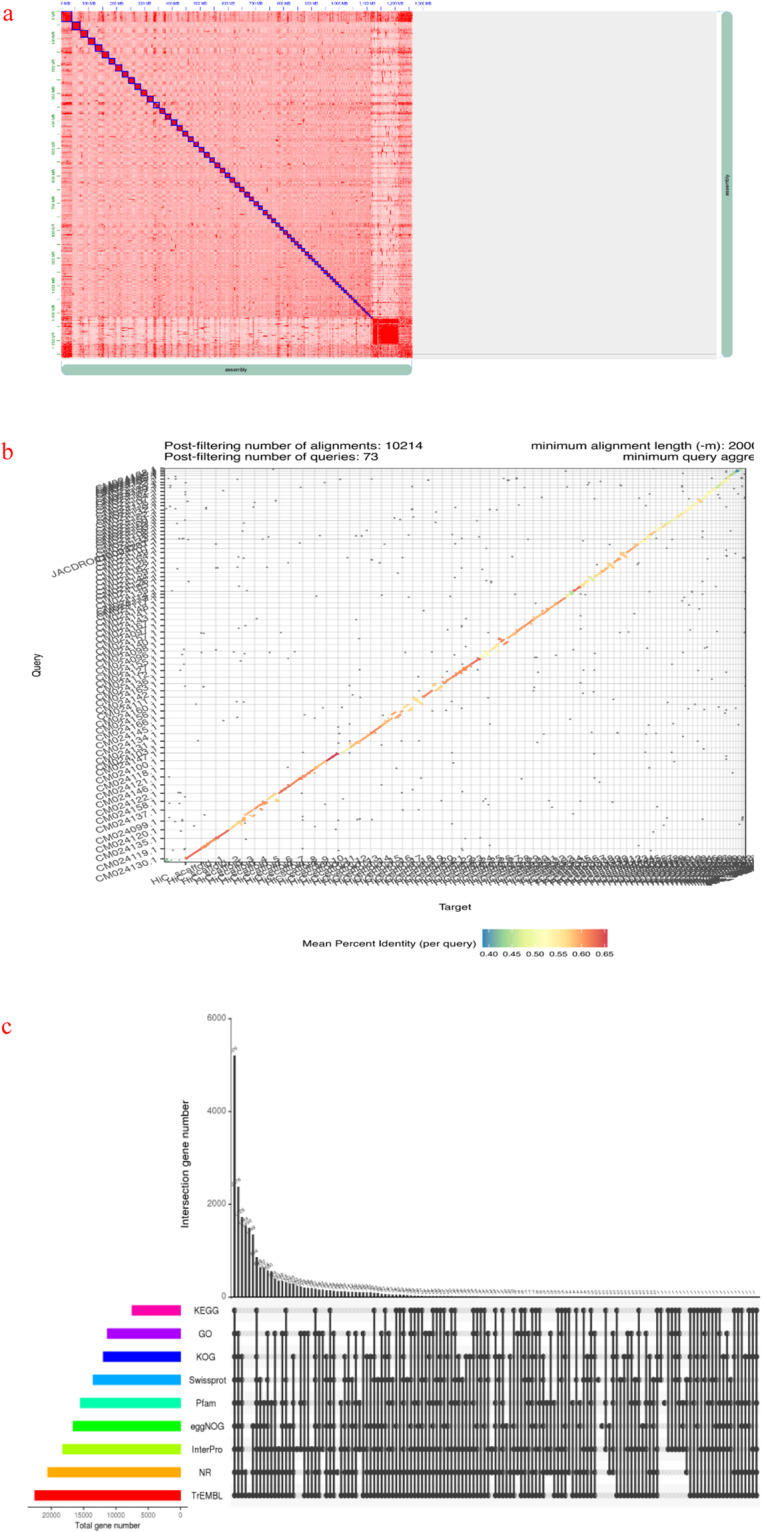
(**a**) Genome-wide HiC interaction heat map of crabs; (**b**) Collinearity analysis of crab and published Chinese mitten crab; (**c**) Gene annotation checkerboard.

With the above Clean data as input, through the juicer^21^ rocess, the default parameters of bwa mem^22^ R1 and R2 were used to compare the genome after PacBio assembly and purge, and the default parameters of 3D-DNA^23^ were used to cluster chromosomes according to the interaction information provided by HiC data. After sorting and two rounds of error correction, the HiC interaction matrix was imported into juicebox for visualization and manual check. After it was confirmed that there was no abnormality, the HIC interaction matrix was exported. 500 N was added between each contig, and the final chromosome mount rate was 87.79%. scaffoldN50 was 17,505,766 bp(approximately 17.50 M) and the genome statistics are given below (Table 3). As you can see, the current results can clearly distinguish 71 chromosome groups (crabs have 72 chromosomes). Within each group, it can be seen that the intensity of the interaction at the diagonal position is higher than that at the non-diagonal position, indicating that the interaction intensity between adjacent sequences (diagonal position) is high, but the interaction signal intensity between non-adjacent sequences (non-diagonal position) is weak, which is consistent with the principle of Hi-C assisted genome assembly. We compared the results of HiC linkage with the published genome of Eriocheir sinensis, and the results showed that there was a good collinearity between the two (Fig. 2a,b).

**Table 3:** HiC assisted assembly N50 statistics.

Species	ScaN um	CtgN um	BaseNum(bp)	MaxCtgLen (bp)	MeanCtgLen (bp)	ScaN50	CtgN50
crab	6911	18497	1,269,461,104	41,762,172	183,687	17,505,766	158,962

### Comparative genomic analysis

**Fig. 3:**
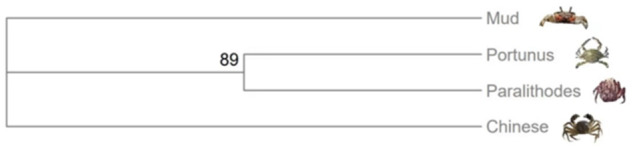
Phylogenetic evolutionary tree of four species.

To enhance our comprehension of the acquired data, we conducted a comparative analysis between the pertinent genomic data we obtained and the genomic data synthesized in analogous published articles^24–26^ (Fig. 3). These articles encompass not only the Chinese mitten crab (Eriocheir sinensis) examined in our study but also the Portunus trituberculatus, the blue king crab, and the mud crab, which exhibit close phylogenetic relationships with the Chinese mitten crab. The phylogenetic tree unequivocally demonstrates that both crab species diverge from a common branch, indicating their close affinity to the Chinese mitten crab. Hence, conducting a comparative analysis of the genomes of closely related species can provide a more comprehensive understanding of their developmental and adaptive evolutionary interconnections.

Additionally, the comparison results (Table 4) reveal that the assembly is in contig form, with a significantly higher number of genes and proteins compared to other datasets. This suggests that our data encompass a broader range in terms of data processing, rendering the results more reliable. Moreover, the completeness of BUSCO is also observed to be the highest, indicating the superior completeness of the assembled genes. The range of other data sets falls within an appropriate range, indicating the significant reference value of our data. However, in terms of total assembly length, our data set exhibits the lowest value among the various sets. This characteristic provides an advantage in obtaining additional information regarding the shortest length, highlighting the potential of our genome and third-generation transcriptome sequencing for studying the relationship between similar species.

**Table 4:** Comparative analysis diagram of different articles’ genomes.

Item	OE assembly	Cui *et al*., 2021	Tang *et al*., 2020	Jun Wang *et al*., 2022
Contig	18,472	1,70,724	6,666	4,808
Contig	1,59,228	26,045	27,47,658	7,17,335
Longest Contig(bp)	30,61,231	14,57,336	1,50,47,233	45,379, 147
Scaffold No.	6,908	1,01,205	4,311	2, 160
Scaffold N50 (bp)	17,444, 176	17, 127,685	1,76,08,299	1,69,75,517
Longest Scaffold(bp)	4,16,92,756	5,08,64,308	3,14,80,327	45,379, 147
BUSCO(%)	96	86	86	95
Gene No.	30, 188	28,033	22,619	20,286
Protein No.	30,661	28,033	22,619	20,286
Repeat (%)	50	45	61	60
Chromoso me No.	71	73	72	70
Total length(bp)	1,26,60,92,319	1,56,76,15,418	1,272, 135, 1	17,67,84,644
Sequencing Data	Illumina(PE150,262 G) PacBio Sequel II(113 G) Hi-C(288 G)	Illumina(PE + MP,374 G) PacBio Sequel II(51 G) Hi-C(132 G) 10xGenomics(PE150,154 G)	Illumina(96 G from Song *et al*., 2016) Oxford Nanopore(53 G)	Illumina(81.2 G) Oxford Nanopore(81.7 G) BioNano(442.0 G) Hi-C(300.5 G)

### The full-length sequences of *E. sinensis* using PacBio sequencing

**Fig. 4:**
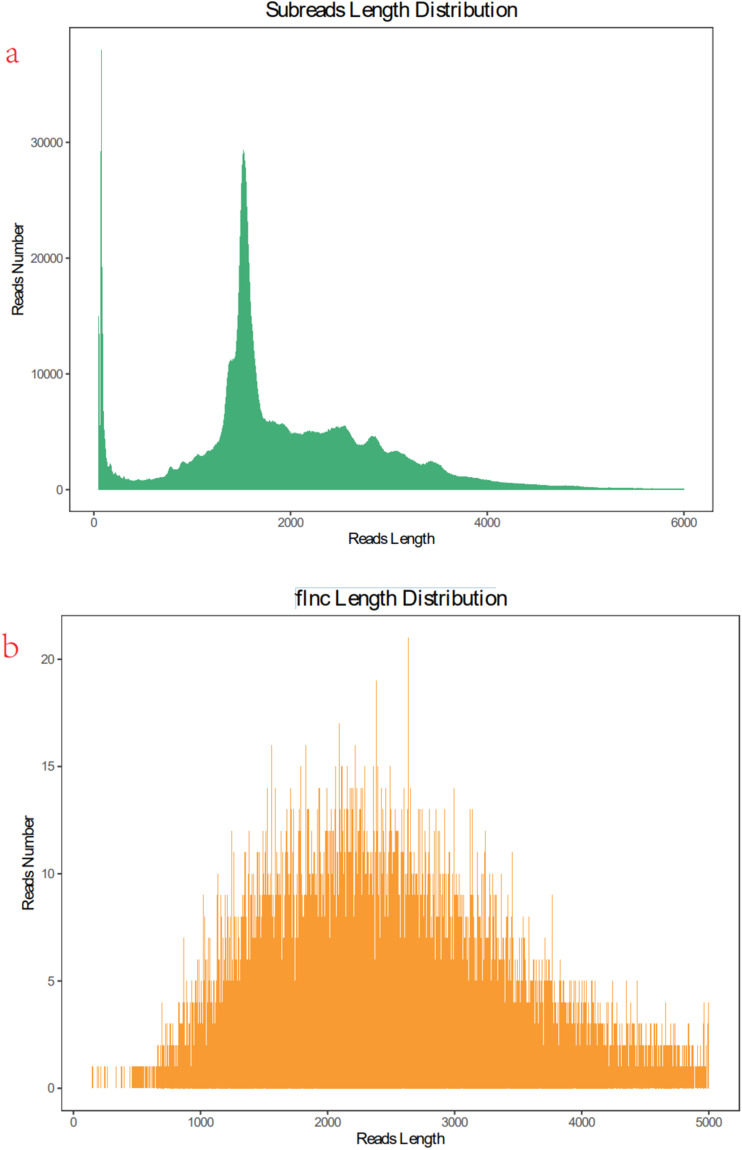
Length Distribution.

**Fig. 5:**
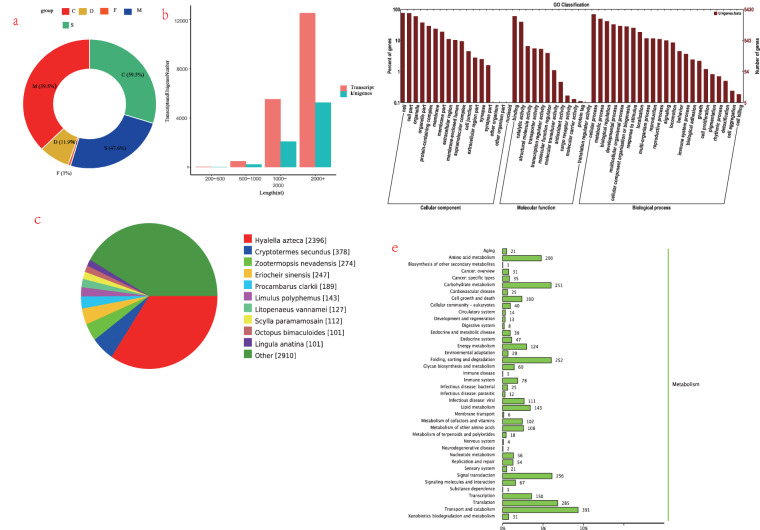
(**a,b**) BUSCO, Transcripts and UnigenesNumber; (**c**) Nr Homologous Species Distribution; (**d**) Go Classification; (**e**) KEGG Classification.

The complete transcriptome of the crab was obtained by utilizing the PacBio Sequel platform to sequence the combined RNA samples from the four major tissues, namely muscle, brain, eyestalk, and heart tissue. This resulted in a total of 36.93 G sub-fragments, from which 175,90041 effective insert fragments were generated. These fragments have an average length of 2099 bp and originate from a single template, ensuring greater accuracy of the information. The lengths of the transcripts N30, N50, and N90 are 3085 bp, 2441 bp, and 1440 bp, respectively. Based on the subread length distribution diagram, it is evident that the fragment length distribution is approximately 1800 bp (Table 5 and Fig. 4a). Out of the 36.93GB sub-reads generated, a total of 511,828 non-chimeric cyclic consistency (CCS) reads were classified, consisting of 394,650 full-length readings and 117,178 non-full-length readings. The consensus sequence length ranges from 200 bp to 5000 bp, with an average length of 2300 bp (Fig. 4b), conforming to the expected unimodal distribution. Subsequently, the consensus sequence is corrected and referred to as Transcripts to differentiate it from the original sequence. A total of 18,539 transcript sequences were acquired, with a predominant concentration of fragment lengths exceeding 2000 base pairs (bp) and exhibiting high repetitiveness. Upon conducting subsequent comparisons, it was observed that an increase in transcript length corresponded to a higher count of transcripts. Subsequently, the CD-HIT-EST tool was employed to cluster the data, resulting in the acquisition of 7545 non-redundant crab transcripts. The length distribution of these transcripts, devoid of any redundancy, was primarily concentrated above 2000 bp. To assess the reliability of multi-tissue transcript sequencing, we employed the Benchmark Universal Single Copy Homologous Sequence (BUSCO) evaluation (Fig. 5a). The clustering results revealed that approximately 70% of both Transcripts and Unigene fragment lengths were concentrated above 2000bp, with the number of intervals increasing as the fragment length increased. Furthermore, Transcripts exhibited superiority in terms of both quantity and length. Specifically, we obtained a total of 18,535 Transcripts sequences and 7,545 Unigenes sequences (Fig. 5b). The number of sequences increased proportionally with their length, with many sequences exceeding 2000nt.

**Table 5:** Description of *E. sinensis* by PacBio Sequel platform.

Sub-fragments	36.93 G
Effective insert fragments	17,590,041
N30(bp)	3085
N50(bp)	2441
N90(bp)	1440
CCS reads	5,11,828
Full-length readings	394650
Non-full-length readings	117178
Non-redundant transcripts	7545
Transcripts sequences	18,535
Unigenes sequences	7545

### Gene structure analysis

**Fig. 6:**
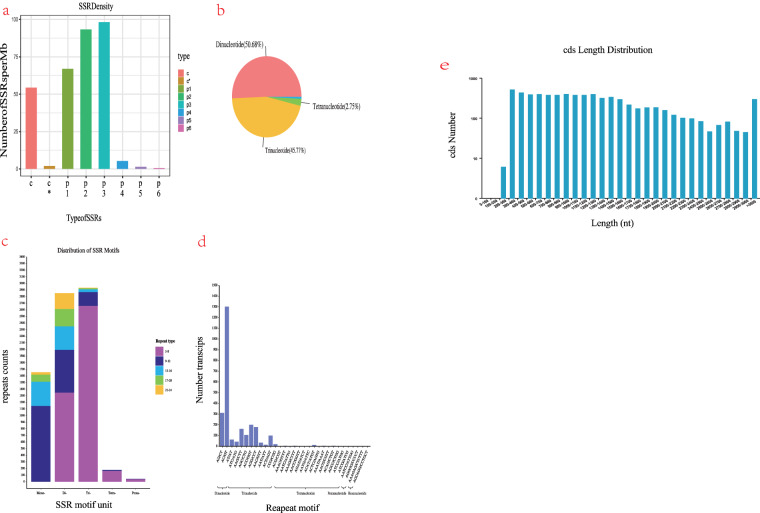
(**a,b**) Number of SSRs per Mb; (**c**) Disstribution of SSR Motifs; (**d**) Number of Repeat Motifs; (**e**) CDS Length Distribution.

The analysis of CDS prediction based on the obtained full-length transcriptome sequences reveals that the majority of CDS lengths fall within the range of 200 to 3000 nt (Fig. 6e). In our investigation, we employed MISA software to examine redundant Unigene sequences exceeding a length of 1 kb. A total of 7,319 sequences were subjected to evaluation, encompassing a cumulative base count of 19,660,022. Within these sequences, 8124 simple sequence repeats (SSR) were identified, with only 3794 sequences containing SSR structures. Notably, half of the SSR-containing sequences exhibited multiple SSRs, while the remaining half manifested as complex combinations. There exist six distinct categories of SSRs, with each sequence exhibiting a unique SSR category. Among these categories, the two-base repeat and three-base repeat SSRs are the most prevalent, collectively constituting 76% of the total occurrences. Conversely, the six-base repeat SSR exhibits the lowest frequency, appearing only ten times. Furthermore, variations in base types persist across different sequences, resulting in limited structural similarities. From the various density distribution maps, it is evident that the frequency of double-base repeats and three-base repeats per Mb is higher compared to four-base repeats, five-base repeats, and six-base repeats. The number of these repeats within each Mb is notably lower (Fig. 6a,b). Furthermore, apart from examining the redundant Unigene sequence, we have also chosen several genes from the Transcript sequence for investigation. Consequently, we have selected genes with identification numbers 5042, 6051, 6052, and 5043 for further analysis. The base lengths of these entities are approximately 3000 bp, exhibiting minimal variation among them. Notably, the SSR types they encompass consist of both Double base repeat and three base repeat patterns (Fig. 6c,d). A meticulous examination reveals a distinctive characteristic in the selected gene ids: the presence of continuous repetitive sequences within a specific length, typically ranging around 20 bases. The initial occurrence of the repetitive sequence is primarily observed within the middle and late segments, thereby exerting no discernible impact on gene transcription and expression prior to the repetitive sequence. The majority of repetitive sequences manifest in a consecutive manner, consisting of two bases, although there exists an occasional occurrence of three consecutive bases within a specific gene. Notably, the GT sequence emerges as the most prevalent repetitive sequence, while the CTC sequence exhibits the lowest frequency.

Long-Chain Non-coding RNA (Long-ChainNonCodingRNA) refers to a specific type of RNA molecule characterized by a transcript length exceeding 200 nt and lacking protein-coding capability. In the context of predicting the coding potential of incomplete and antisense transcripts, CNCI demonstrates efficacy, while CPC utilizes support vector machine classifiers for evaluating transcript coding potential. Additionally, Pfam employs hmmscan homology search to predict coding potential. In the crab transcriptome, the three aforementioned methods identified a total of 5349, 1389, and 2014 lncRNAs, respectively. The intersection of these three results yielded 1207 lncRNA transcripts.

### Gene structure prediction

Repeat sequences can be divided into two categories: Tendam repeat and Interpersed repeat. The series repeat sequences include microsatellite sequences, small satellite sequences, etc. Scattered repeat sequences, also known as transposon elements, include DNA transposons and retrotransposons that transpose in a DNA-DNA manner. Common retrotransposon classes include LTR, LINE and SINE. We use the EDTA^27^ process, which calls software such as LTR_finder^28^, LTRdetector^28^, HelitronScanner, RepeatMasker and RepeatModeler(Smit, A. F., & Hubley, R. (2008). RepeatModeler Open-1.0. Available fom http://www.repeatmasker.Org). After obtaining the repeat sequence, in order to verify the accuracy of the prediction of the repeat sequence, the predicted repeat sequence in the genome was replaced with N (that is, hard mask), and then BUSCO integrity assessment was performed. The results showed that there were 49.83% repeats in the crab genome, among which the proportion of scattered repeats was 34.68% and the proportion of tandem repeats was 15.14%. BUSCO assessment of the genome after hard mask showed that the completeness was 94.4%. Before, the integrity of the genome level was 95.6%. After masking the repeat sequence, the integrity decreased slightly, but the decline was within the acceptable range (Table 6). It shows that the repeated sequence predicted by us is accurate. Gene structure prediction includes homology prediction and de novo prediction. Homologous prediction we mainly rely on RNA-Seq data and protein information of closely related species, referring to published articles, The homolog species were Chinese eriocheirus (Eriocheir_sinensis), white shrimp (Penaeus_vannamei), Portunus_trituberculatus, and bumble bees (Bombus_terrestris). We used hisat2^29^ to compare the RNA-Seq data to the genome, and StringTie to guide the genome assembly. We used MAKER^30^ process to compare the assembled transcripts and homologous proteins of related species to the reference genome using blastn/blastx and exonerate to perform the first round of gene prediction and select AED &lt; 0.1 of the gene trains SNAP. At the same time, we trained Augustus and GeneMark-ES by braker, using RNA-Seq and homologous protein data of closely related species. Finally, using MAKER^30^ software again, we integrated the first round of homology prediction evidence and invoked Augustus^31^, SNAP^32^, and GeneMark-ES^32^ for the second round of de novo gene prediction to get the final gene set. Finally, we predicted 30,188 genes and 30,661 protein sequences. The integrity of the protein sequence was 89.7%.

**Table 6:** Crab repeat sequence statistics.

Class	Count	bpMasked	%masked
DNA	—	—	—
DTA	235503	62691279	4.95%
DTC	293645	46129784	3.64%
DTH	45599	7559054	0.60%
DTM	331286	63515079	5.02%
DTT	92040	13557745	0. 17%
Helitron	299447	43008315	3.40%
LTR	—	—	—
Copia	256	77925	0.01%
Gypsy	167634	55434958	4.38%
unknown	352825	135781543	10.72%
MITE	—	—	—
DTA	38055	4960868	0.39%
DTC	10370	1097092	0.09%
DTH	12186	1234624	0. 10%
DTM	27617	4031807	0.32%
DTT	415	34301	0.00%
total interspersed	1906878	439114374	34.68%
Low_complexity	255669	30522569	2.41%
Simple_repeat	1776544	161175759	12.73%
Total	3939091	630812702	49.83%

### Gene function annotation

Gene functional annotations include NR annotations, COG/KOG functional annotations, GO classification, Swiss-Prot, TrEMBL, eggNOG, KEGG, InterPro, and Pfam. For NR, COG/KOG, GO, Swiss-Prot, TrEMBL, eggNOG and KEGG database annotations, diamond^33^ software was used for comparison and e&lt was obtained. 1e-5 annotation, screening for proteins with the highest sequence similarity, so as to obtain functional annotation information. InterProscan^34^ (v5.36.75) annotates proteins to Pfam and InterPro databases based on their domains and motif elements. (Table 7 and Fig. 2c).

**Table 7:** Comment rate statistics for each databases.

#Anno_Database	Annotated_Number	Annotated_percent
NR	20519	66.92%
Swissprot	13522	44.10%
TrEMBL	22535	73.50%
KEGG	7472	24.37%
KOG	11927	38.90%
eggNOG	16603	54.15%
GO	11318	36.91%
Pfam	15483	50.50%
InterPro	18224	59.44%

To achieve a comprehensive functional annotation of the crab transcriptome, we utilized six databases (Swiss-Prot, KOG, GO, NR, Pfam, and KEGG) to annotate a total of 7033 non-redundant transcripts. Among these, 7004, 5880, 6884, 5308, 5430, and 5684 transcripts were successfully marked. When comparing the main species distribution against the NR database, the majority of the matched transcripts exhibited similarity to *Hyalella Azteca* (2396), *Cryptotermes secundus* (378), *Zootermopsis nevadensis* (274), *Eriocheir sinensis* (2470), *Procambarus clarkii* (189), *Limulus polyphemus* (143), *Litopenaeus vannamei* (127), *Scylla paramamosain*(112), *Octopus bimaculoides* (101), *Lingula anatine*(101) and other species (2910) (Fig. 5c). In the BLASTX analysis of NR protein hits, the annotation results revealed a significant proximity between the majority of transcripts and genes from other crustaceans. As anticipated, these transcripts exhibited the highest frequency of hits with amphibians (2396 occurrences, accounting for 33.7% of the total), followed by cryptotermites (378 occurrences, representing 5.4%). Notably, 2910 transcripts exhibited no homologous sequences in publicly available databases.

The GO database was employed to annotate the complete transcripts of crabs, resulting in the successful categorization of 5430 transcripts into three distinct categories: biological process, molecular function, and cellular component. The majority of the annotated transcripts exceed a length of 1000 base pairs. Within the realm of biological processes, cellular processes constitute the largest proportion, followed by metabolic processes and biological regulation. Furthermore, our analysis revealed the annotation of certain genes pertaining to distinct biological processes, such as biological process regulation, positioning, response to stimuli, and signal terms. Among the cellular components, the genes associated with cells, cell parts, organelles, membranes, membrane parts, and macromolecular complexes exhibit the highest representation. In the category of molecular function, the terms binding, catalytic activity, and transporter activity exhibit the highest frequency (Fig. 5d). By means of KOG analysis, a total of 5308 transcripts were annotated and classified into 26 KOG categories. The preeminent cluster among these categories is R, which consists of genes that predict general function. Following R, the T cluster comprises genes involved in signal transduction mechanisms, indicating that the majority of functions represented by these transcripts are associated with the regulation of cell growth, proliferation, metabolism, and various other functional mechanisms. The third category encompasses O (post-translational modification, protein renewal, molecular chaperones), Z (cytoskeleton), and S (unknown function). The smallest cluster, N, pertains to cell migration.

The KEGG pathway analysis method enables a comprehensive examination of the metabolic pathways of gene products and compounds within cellular systems, as well as the functional roles of these gene products. Within KEGG’s classification scheme, crabs are categorized into various groups, with human diseases, metabolism, and body systems emerging as the most prominent categories, collectively representing a substantial proportion. Specifically, the human disease-related pathways encompass a total of 359 genes, wherein 111 genes are implicated in infectious diseases caused by viruses, 25 genes are associated with infectious diseases caused by bacteria, and 31 genes are linked to cancer-related processes. In brief, this study identifies a comprehensive set of 242 genes that are categorized into various biological systems pathways, namely the nervous system (4 genes), immune system (78 genes), digestive system (8 genes), and endocrine system (47 genes). Notably, these four pathways exhibit the highest gene abundance. Furthermore, an additional 1094 annotated genes are found to be implicated in metabolic pathways. The pathways exhibiting the highest abundance are carbohydrate metabolism (251 genes) and amino acid metabolism (200 genes). Regarding environmental information processing, genes associated with signal transduction (256 genes), signal molecules and their interactions (67 genes), and membrane transport (6 genes) are prevalent. Conversely, a lower number of genes are annotated for cellular processes and genetic information processing (Fig. 5e).

### Comparative analysis of gene functional annotation in genome and transcriptome

The process of genome annotation encompasses three primary components, namely the annotation of repeat sequences, gene annotation (encompassing both gene structure prediction and gene function prediction). In this particular investigation, gene function annotations were conducted on both the genome and transcriptome of the third generation. Consequently, a comparative analysis was performed based on the disparities in their annotations, aiming to enhance comprehension of crab’s gene function and establish a foundational basis for subsequent research and analysis in this field. The functional annotation of the genome involved the utilization of the diamond software^33^ for comparative analysis. A threshold of e < 1e-5 was employed to identify proteins with the greatest sequence similarity, thereby obtaining functional annotation information. InterProscan (v5.36.75)^34^ was utilized to annotate proteins to the Pfam and InterPro databases, utilizing their respective domains and motif elements. Notably, the TrEMBL database exhibited the highest proportion of database comments (73.50%), while the KEGG database displayed the lowest (24.37%). To achieve comprehensive annotation of the transcriptome, a total of 7033 non-redundant transcripts were annotated utilizing six databases, namely Swiss-Prot, KOG, GO, NR, Pfam, and KEGG. The gene function of the relevant cell of the river crab was successfully elucidated in the majority of these databases. However, in the NR database, a significant proportion of the analyzed transcripts exhibited close similarity to genes of other crustaceans, with the highest accuracy observed in amphibians (2396 instances, accounting for 33.7%), followed by cryptotermites (378 instances, accounting for 5.4%). These data may partially elucidate alterations in the evolution of the crab gene family. Consequently, both genome and transcriptome functional annotation analysis can offer a limited comprehension of cellular gene function in crabs. However, the analysis of the three-generation transcriptome provides a more comprehensive and specific examination compared to genome functional analysis, and holds greater significance as a point of reference.

## Data Records

The sequencing data have been archived in the National Center for Biotechnology Information (NCBI)^35^, and more detailed data on survey, PacBio, Genome purge, HiC, RNAseq, and ISO-seq are also included. Finally, the whole genome sequencing and annotation data are stored at NCBI’s GenBank: JAWQET000000000^36^, and Figshare^37^.

## Technical Validation

In order to guarantee the integrity of the sequencing data, state-of-the-art molecular biology equipment was employed to assess the purity, concentration, and integrity of RNA samples intended for RNA sequencing. The selected sample for this study exhibited an RNA integrity number (RIN) exceeding 8.5. The primary protocols employed in libraries were as follows: (1) NEBNext Single Cell/Low Input cDNA Synthesis & Amplification Module was utilized to synthesize the complete mRNA cDNA; (2) Full-length cDNA underwent PCR amplification; (3) Repair and terminal repair of full-length cDNA were conducted; (4) SMRT dumbbell connector was employed for library construction. Subsequently, the library’s quality was evaluated through testing. Once the requirements are met, computer sequencing can be conducted. Following the successful completion of the library inspection, the PacBio instrument is utilized to sequence the complete transcriptome based on the desired data volume. Employing the PacBio Sequel II platform, the original data of approximately 113 G (113,241,560,168 bp) is sequenced, with a genome size of approximately 1.4 G (1,486,013,762 bp) and a total of 1,929,604,516 bp in reads. In total, 288,759,220,191 bp of Hi-C data is generated, with Q20 and Q30 values of 96.26% and 90.85% for the Hi-C reads, respectively.

## Data Availability

No custom code was used to generate or process the data described in this manuscript.
